# Management of complicated diaphragmatic hernia in the acute setting: a WSES position paper

**DOI:** 10.1186/s13017-023-00510-x

**Published:** 2023-07-26

**Authors:** Mario Giuffrida, Gennaro Perrone, Fikri Abu-Zidan, Vanni Agnoletti, Luca Ansaloni, Gian Luca Baiocchi, Cino Bendinelli, Walter L. Biffl, Luigi Bonavina, Francesca Bravi, Paolo Carcoforo, Marco Ceresoli, Alain Chichom-Mefire, Federico Coccolini, Raul Coimbra, Nicola de’Angelis, Marc de Moya, Belinda De Simone, Salomone Di Saverio, Gustavo Pereira Fraga, Joseph Galante, Rao Ivatury, Jeffry Kashuk, Michael Denis Kelly, Andrew W. Kirkpatrick, Yoram Kluger, Kaoru Koike, Ari Leppaniemi, Ronald V. Maier, Ernest Eugene Moore, Andrew Peitzmann, Boris Sakakushev, Massimo Sartelli, Michael Sugrue, Brian W. C. A. Tian, Richard Ten Broek, Carlo Vallicelli, Imtaz Wani, Dieter G. Weber, Giovanni Docimo, Fausto Catena

**Affiliations:** 1 General Surgery Unit, Maggiore Hospital, Parma, Italy; 2 Department of Emergency Surgery, Maggiore Hospital, Via A. Gramsci 14, 43126 Parma, Italy; 3 Research Office, College of Medicine and Health Sciences, UAE University, Al-Ain, United Arab Emirates; 4 Emergency and Trauma Surgery, Bufalini Hospital, Cesena, Italy; 5 Department of General Surgery, IRCCS Policlinico San Matteo Foundation, Pavia, Italy; 6 General Surgery, Department of Clinical and Experimental Sciences, University of Brescia, Brescia, Italy; 7 John Hunter Hospital, University of Newcastle, Newcastle, NSW Australia; 8 Acute Care Surgery at The Queen’s Medical Center, John A. Burns School of Medicine, University of Hawai‘I, Honolulu, USA; 9 Department of General and Foregut Surgery, IRCCS Policlinico San Donato, University of Milano, Milan, Italy; 10 Healthcare Administration, Santa Maria Delle Croci Hospital, AUSL Romagna, Ravenna, Italy; 11 Department of Morphology, Surgery and Experimental Medicine, University Hospital of Ferrara and University of Ferrara, Ferrara, Italy; 12 General and Emergency Surgery, School of Medicine and Surgery, Milano-Bicocca University, Monza, Italy; 13 Department of Surgery and Obstetrics/Gynaecology, Regional Hospital, Limbe, Cameroon; 14 General, Emergency and Trauma Surgery Department, Pisa University Hospital, Pisa, Italy; 15 Riverside University Health System Medical Center, , Riverside, California, USA; 16 Unit of Colorectal and Digestive Surgery, DIGEST Department, Beaujon University Hospital, AP-HP, University of Paris Cité, Clichy, France; 17 Trauma/Acute Care Surgery, Department of Surgery, Medical College of Wisconsin, Milwaukee, WI USA; 18 Department of General and Metabolic Surgery, Poissy and Saint‐Germain‐en‐Laye Hospitals, Poissy, France; 19 Department of General Surgery, San Benedetto del Tronto General Hospital, San Benedetto del Tronto, Italy; 20 Division of Trauma Surgery, Department of Surgery, School of Medical Sciences, University of Campinas (Unicamp), Campinas, SP Brazil; 21 Trauma Department, University of California, Davis, Sacramento, CA USA; 22 Department of Surgery, Virginia Commonwealth University School of Medicine, Richmond, VA USA; 23 Department of Surgery, Sackler School of Medicine, Tel Aviv University, Tel Aviv, Israel; 24 Department of General Surgery, Albury Hospital, Albury, Australia; 25 Department of General, Acute Care, Abdominal Wall Reconstruction, and Trauma Surgery, Foothills Medical Centre, Calgary, AB Canada; 26 Department of General Surgery, Division of Surgery, Rambam Health Care Campus, Haifa, Israel; 27 Department of Primary Care and Emergency Medicine, Kyoto University Graduate School of Medicine, Kyoto, Japan; 28 Abdominal Center, University Hospital Meilahti, Helsinki, Finland; 29 Department of Surgery, University of Washington, Seattle, WA USA; 30 Department of Surgery, Denver Health Medical Center,, University of Colorado, Denver, CO USA; 31 University of Pittsburgh School of Medicine, Pittsburgh, PA USA; 32 General Surgery Department, Medical University, University Hospital St George, Plovdiv, Bulgaria; 33 Department of Surgery, Macerata Hospital, Macerata, Italy; 34 Department of Surgery, Letterkenny University Hospital, Letterkenny, Donegal Ireland; 35 Department of General Surgery, Singapore General Hospital, Singapore, Singapore; 36 Surgery Department, Radboud University Medical Center, Nijmegen, The Netherlands; 37 Department of Minimal Access and General Surgery, Government Gousia Hospital, Srinagar, India; 38 Department of Trauma Surgery, Royal Perth Hospital, Perth, Australia; 39 Department of Medical and Advanced Surgical Sciences, University of Campania “Luigi Vanvitelli”, Naples, Italy

**Keywords:** Diaphragm hernia, Emergency surgery, Guidelines, Rupture, Trauma, Congenital

## Abstract

**Background:**

Diaphragmatic hernia (DH) presenting acutely can be a potentially life-threatening condition. Its management continues to be debatable.

**Methods:**

A bibliographic search using major databases was performed using the terms “emergency surgery” “diaphragmatic hernia,” “traumatic diaphragmatic rupture” and “congenital diaphragmatic hernia.” GRADE methodology was used to evaluate the evidence and give recommendations.

**Results:**

CT scan of the chest and abdomen is the diagnostic gold standard to evaluate complicated DH. Appropriate preoperative assessment and prompt surgical intervention are important for a clinical success. Complicated DH repair is best performed via the use of biological and bioabsorbable meshes which have proven to reduce recurrence. The laparoscopic approach is the preferred technique in hemodynamically stable patients without significant comorbidities because it facilitates early diagnosis of small diaphragmatic injuries from traumatic wounds in the thoraco-abdominal area and reduces postoperative complications. Open surgery should be reserved for situations when skills and equipment for laparoscopy are not available, where exploratory laparotomy is needed, or if the patient is hemodynamically unstable. Damage Control Surgery is an option in the management of critical and unstable patients.

**Conclusions:**

Complicated diaphragmatic hernia is a rare life-threatening condition. CT scan of the chest and abdomen is the gold standard for diagnosing the diaphragmatic hernia. Laparoscopic repair is the best treatment option for stable patients with complicated diaphragmatic hernias. Open repair is considered necessary in majority of unstable patients in whom Damage Control Surgery can be life-saving.

## Background

Complicated diaphragmatic hernia (DH) is a rare life-threatening condition. It is a defect in the diaphragm or its attachments having the risk of the protrusion of the abdominal contents into the thoracic cavity. It can be congenital or acquired. Acquired DH usually occurs following trauma. It occurs in 1–5% of victims of vehicle crashes and in 10–15% of penetrating injuries of the lower chest. Complicated congenital diaphragmatic hernias are less frequent [1–4].

Complicated DH can be associated with large herniation of intra-abdominal organs that may cause incarceration, perforation or strangulation of the organs. The herniation of the abdominal contents into the chest may compress the lungs causing respiratory failure or compressing the heart causing cardiac tamponade. However, the diagnosis of a diaphragmatic hernia can be difficult and delayed. It can be easily missed in the acute setting due to its rarity, nonspecific findings and not being thought of. There is a paucity of clinical guidelines to help the acute care physicians to diagnose and manage this condition. This is the first position paper on this topic aiming to guide clinicians to diagnose and manage diaphragmatic hernias in the emergency setting [5–7].

## Methods

The present paper has been developed according to the GRADE methodology [8, 9].

An extensive bibliographic search of the literature was performed including Medline, EMBASE and PubMed in order to identify articles on complicated diaphragmatic hernias covering the period of January 1983 up to December 2022. The search strategy was conducted identifying articles reporting the item “diaphragmatic hernia,” and then, it has been meshed using the Boolean operator “AND” and “OR” with the following mesh terms: “traumatic diaphragmatic rupture,” “congenital diaphragmatic hernia,” “acquired diaphragmatic hernia,” “trauma,” “lesion,” “rupture” and “emergency surgery.” Additional articles were searched by manual identification from the key articles. No other search restrictions were imposed except the studied period. This allowed identification of published abstracts of clinical trials, comparative studies, congresses, guidelines, government publications, multicenter studies, systematic reviews, meta-analysis, case series, original articles, randomized controlled trials and case reports.

Two researchers (M.G. and G.P) coordinated by a central coordinator (F.C) developed the questions and assembled the different answers to cover the field of this pathology.

Leading specialists in the field then were asked to revise the manuscript. Through subsequent rounds, the expert’s opinions were included in the manuscript. Expert group discussed the definitive version. The final version after agreement was reached resulted in the present manuscript.

### Classification of diaphragmatic hernia

DH is typically classified into congenital diaphragmatic hernias (CDHs) or acquired diaphragmatic hernias (ADHs) Table 1.

**Table 1:** Diaphragmatic hernia classification

Congenital diaphragmatic hernia (CDH)
Bochdalek hernias: posterior left side of diaphragm, 85% of the cases
Morgagni hernia: anterior, retrosternal or parasternal defect of the diaphragm
Diaphragmatic eventration: anteromedial portion of the right hemidiaphragm
Central tendon defects
Acquired diaphragmatic hernia (ADH)
Hiatal hernia: types I–IV, sliding (90%) or paraesophageal hernias (10%)
Traumatic: Blunt or penetrating
Iatrogenic

### Congenital diaphragmatic hernia in adults

CDHs are rare and usually occur on the left side (80%) of the diaphragm. Normally, the diaphragm forms and divides the thoracic cavity from the abdominal cavity during the eighth week of gestation. CDH occurs from incomplete development of the diaphragm. CDH in childhood becomes symptomatic when there is pulmonary hypoplasia. This is caused by the presence of the abdominal organs in the chest cavity during the prenatal period which prevents or delays the lung development. [10–12]

*Bochdalek hernia (BH)* is the most common CDH (95%), occurring more commonly on the posterior left side of the diaphragm (85%, versus right side 15%) [13]. BH in the adult patient has an incidence of 0.17% presenting at an average age of 40 years. Its diagnosis is difficult because of its rarity and its wide range of presenting symptoms. Failure to promptly diagnose and treat a symptomatic BH may lead to complicated BH in up to 25% of cases [10, 14–16]. *Morgagni hernia* (MH) was first described by Morgagni in 1769 as an anterior, retrosternal or parasternal defect of the diaphragm [17] which develops from failure of the complete migration of muscle fibers to cover the triangular space located between the sternum and the bilateral costal margins (foramina of Morgagni). It accounts for approximately 2% of all CDH. MH can remain asymptomatic, and therefore, its diagnosis is often incidental [18, 19]. In a retrospective study, it was diagnosed at an average age of 42 years. Complicated MH can occur in up to 10% of cases [20]. *Diaphragmatic eventration* occurs in the anteromedial portion of the right hemidiaphragm because of incomplete muscularization of the diaphragm which is replaced by a thin membranous sheet [21]. *Central tendon defects* of the diaphragm are extremely rare and are usually associated with significant pericardial effusion and lung hypoplasia [22].

### Acquired diaphragmatic hernia

ADH occurs usually in adulthood. ADH can be categorized into 3 types: (1) hiatal, (2) traumatic and (3) iatrogenic hernias.

### Hiatal hernia

Hiatal hernias are included into acquired diaphragmatic hernias classification, but they are not real diaphragmatic hernia. They can be classified into four main types (1) Type I is a sliding hiatal hernia. It is the most common type (90%) and occurs when the esophageal hiatus is widened enough to allow herniation of the gastric cardia resulting in the migration of the gastroesophageal junction to be above the diaphragm [23], (2) Type II is a paraesophageal hiatal hernia and accounts of 10% of the hiatal hernias. The defect is in the phrenoesophageal membrane that allows the herniation of the gastric fundus, while preserving the gastroesophageal junction in a normal position [24], (3) Type III is the combination of a Type I and Type II hiatal hernias; it is a paraesophageal hiatal hernia with superiorly displaced gastroesophageal junction [23] and (4) Type IV is a significantly large diaphragmatic hernia that can accommodate the herniation of additional viscera including the stomach, colon and spleen.

### Traumatic diaphragmatic hernia

Traumatic diaphragmatic hernias (TDHs) are caused by a traumatic incident that creates a defect in the diaphragm as a result of a sudden increase of intra-abdominal pressure or a penetrating injury. This will cause a tear in the diaphragm and may be associated with migration of abdominal contents from the abdomen to the thoracic cavity. Diaphragmatic rupture occurs in 2.1% of blunt trauma and 3.5% of penetrating trauma [24, 25]. Overall, diaphragmatic rupture (DR) is infrequent and may reach up to 5% of trauma patients depending on the injury severity and mechanism of injury [26].

The mechanism of rupture diaphragm will vary between different countries depending on the prevalence of certain mechanisms. Penetrating trauma is the most frequent mechanism of TDH (65%), but their defects are generally smaller than those following blunt trauma. Currently, the most frequent penetrating thoracic trauma are caused by knife wounds. Blunt trauma accounts for approximately 3–8% of all TDH [27, 28]. The most common cause of blunt diaphragmatic rupture is road traffic collisions. High risk of associated intra-abdominal injuries must be considered in determining the optimal surgical approach requiring an open or laparoscopic approach to evaluate the entire abdomen.

Left-sided injuries are more common after blunt and penetrating trauma in those treated at the hospital due to the protective effect of the bare area of the liver in contact with the diaphragm on the right side, reducing the traumatic force [29]. Those patients will have a better chance of survival. The distribution of DH after blunt trauma is as follows: 50–80% affect the left hemidiaphragm, 12–40% affect the right side, while 1–9% are bilateral [26]. In contrast autopsied victims display predominance on the right side. Aun et al. observed a significantly higher incidence of diaphragmatic lacerations on the right side in an autopsy group (49.6% of 146 cases identified in 12,276 autopsies) compared with the hospitalized group (14.4% of 97 operated cases). However, depending on the mechanism of trauma, both diaphragms can be injured [30–33].

It is important to distinguish between an acute isolated diaphragmatic injury and the potential presence of other associated injuries. Trauma may cause a tiny rupture of the diaphragm, which will remain asymptomatic or may cause delayed complications. This accounts for the typical delayed onset of symptoms of diaphragmatic hernias, few years post-injury [7].

AAST grading of traumatic hernia is reported on Table 2 [34].

**Table 2:** AAST diaphragm injury scale

Grade*	Description of injury
I	Contusion
II	Laceration < 2 cm
III	Laceration 2–10 cm
IV	Laceration > 10 cm with tissue loss (< 25 cm^2^)
V	Laceration with tissue loss > 25 cm^2^

*Advance one grade for bilateral injuries up to grade III

## Clinical presentation

Symptoms of DH will depend on their etiology and can be very insidious. Delayed diagnosis of DH was reported in 5–45% of all CDH [19]. CDH in the adulthood can present with nonspecific respiratory and gastrointestinal symptoms and signs. Severe symptoms have been reported in 46% of CDH [35]. In left-sided CDH, gastrointestinal symptoms including intestinal obstruction are more common, while right-sided CDH predominantly have respiratory symptoms. Mild chronic symptoms (dyspepsia, recurrent or nonspecific abdominal pain) may occur. Occasionally, acute abdominal symptoms caused by bowel obstruction, ischemia, or perforation can cause cardio-respiratory failure [19, 36, 37].

Patients with BH typically develop gastrointestinal (GI) symptoms. These hernias can subsequently be complicated with gastric obstruction, strangulation, volvulus, incarceration, dysphagia, bleeding and respiratory symptoms [38]. Conversely, GI and respiratory symptoms are both common in symptomatic MH in adults. MH has a lower incidence of bowel incarceration or strangulation compared with BH [15].

The symptoms of TDH will depend on the involved side, the severity of the trauma, the type of trauma (blunt or penetrating) and on the amount of energy transferred to the body [39]. The effects of the diaphragmatic rupture are primarily on the circulation and respiration resulting in a 25–50% decrease in the pulmonary function. The most common symptoms are dyspnea (86%) and abdominal pain (17%) [40, 41].

The natural history of traumatic diaphragmatic injury with hernia formation has been described to have three phases (the Carter’s scheme): the acute phase, the latent phase, and the obstructive phase [20]. In the acute phase, between 33 and 66% of TDH are missed [42] because TDHs are often associated with other thoraco-abdominal, cerebral, or musculoskeletal injuries. The associated injuries are responsible for the poor prognosis and not the TDH [42]. In the latent phase, there are nonspecific GI and respiratory symptoms caused by the herniation (sometimes intermittent) of the intra-abdominal viscera into the thoracic cavity [43] In the obstructive phase, visceral obstruction may progress to ischemia of the herniated organs.

Delayed presentation of DH is common. DH can be asymptomatic for decades before becoming symptomatic in later stages. This occurs either because the hernia becomes huge, or it has already caused secondary visceral complications [44]. Nonspecific presentation may lead to misdiagnosis and inappropriate management. Antecedent viral illness with subsequent respiratory distress may lead to the misdiagnosis of pneumonia or bronchiolitis. As the diameter of the diaphragmatic injury increases over time, herniation of the abdominal organs and subsequent worsening clinical symptoms occur [45, 46].

Undetected DH may cause strangulation of the bowel and subsequent perforation causing severe peritonitis, sepsis and multi-organ failure which is a surgical emergency having a high mortality. Commonly, patients with delayed diagnosis have right-sided diaphragmatic ruptures (50%) [48]. In a series of 980 cases, the diagnosis of DH was made preoperatively in 43.5% of the patients, the diagnosis of 41.3% of the patients was reached during autopsy or surgical exploration, and in the remaining 14.6% of the patients, the diagnosis was delayed until the clinical condition worsened [42].

## What is the best way to diagnose DH in the emergency setting?

- In patients without a history of trauma and with respiratory symptoms, a chest X-ray both anteroposterior and lateral is recommended as the first diagnostic study (Strong recommendation based on low-quality evidence, 1C).
- In stable trauma patients with suspected DH (nonspecific symptoms and chest X-ray), CT scan with contrast enhancement of the chest and abdomen is recommended (Strong recommendation based on moderate-quality evidence, 1B).
- In stable trauma patients with lower chest penetrating wounds and suspected DH (nonspecific symptoms and chest X-ray), diagnostic laparoscopy is recommended (Weak recommendation based on low-quality evidence, 1C).
- Endoscopy is not recommended in traumatic hernias (Weak recommendation based on low-quality evidence, 2C).
- In pregnant patients with suspected non-traumatic DH, ultrasonography is suggested as the first diagnostic study (Weak recommendation based on very low-quality evidence, 2D).
- In stable pregnant patients with suspected non-traumatic DH, MRI is suggested after ultrasonography (Weak recommendation based on very low-quality evidence, 2D).

The most commonly used diagnostic test for DH in the literature, but not necessarily the most accurate, is the chest X-ray. However, initial radiographic findings can be misinterpreted in around 25% of the cases [4]. Normal chest radiographs have been reported to range from 11 to 62% in diaphragmatic injuries or in uncomplicated diaphragmatic hernias [40, 49, 50]. Suspicious chest X-ray findings in DH include an abnormal bowel gas pattern, an air-fluid level, an abnormal lucency or soft tissue opacity with deviation of the mediastinum, or hemidiaphragm elevation. Larger hernias may show loops of small or large bowel. When the nature of the thoracic contents is uncertain, a nasogastric tube located inside the herniated stomach can be diagnostic [3]. The chest X-ray has a sensitivity of 2–60% for diagnosing the left-sided hernia and 17–33% for right-sided hernia [51]. Despite advances in imaging methods, the chest X-ray is still useful. It is easy, inexpensive, have low radiation, and universally available. In cases of persistent clinical suspicion, a CT scan should be performed to confirm or refute the diagnosis [52].

CT scan is the gold standard for diagnosing the diaphragmatic hernia. It has a sensitivity and specificity of 14–82% and 87%, respectively [35, 53]. Nevertheless, it may miss small tears of penetrating injuries like stab wounds, when no hernia has yet occurred. Unlike the chest X-ray, which can be normal in intermittent herniation, CT scan is more accurate in determining the presence, location and size of the diaphragmatic defect. Chest and abdominal CT scan can evaluate the intrathoracic complications of the herniated abdominal organs [54]. The various radiological findings which have been described in the literature include: diaphragmatic discontinuity, segmental non-recognition of the diaphragm, “Dangling diaphragm” sign (visualization of the free edge of the ruptured diaphragm curling toward the center of the abdomen away from the chest wall) [55], “Dependent viscera” sign [56] (no space between the liver, bowel or stomach and the chest wall, described as abutted), intrathoracic herniation of abdominal contents, “Collar sign” (constriction of the herniating abdominal organ at the level of the rupture) [57], contiguous injuries of both sides of the diaphragm, elevated abdominal organs, thickened diaphragm, thoracic fluid, abutting intra-abdominal viscera, hypo-attenuated hemidiaphragm and associated fractured ribs [28, 58–60]. CT scan findings of ischemia include forward displacement of the gastric bubble, the missing of the gastric folds, the absence of gastric walls contrast enhancement, intestinal wall thickening with target enhancement, spontaneous hyper-density of the intestinal wall, lack of enhancement after injection of iodinated contrast agent, and parietal pneumatosis with portal and mesenteric venous gas [53, 61].

Laparoscopy and thoracoscopy can be useful in stable patients with a thoraco-abdominal penetrating wound. Small diaphragmatic defects may be not detected by both chest X-ray and CT scan [62–64].

Other diagnostic tests such as esophagogastroduodenoscopy, ultrasound and barium studies have been used in the diagnosis of complicated DH. Endoscopy is useful in stable patients to assess the presence of erosive esophagitis or gastroesophageal reflux especially in patients with hiatal hernia.

Endoscopy can determine the size and type of hernia and evaluate the gastric viability. However, the role of endoscopy in the diagnosis of diaphragmatic hernia, especially in emergency setting, is underreported. Endoscopy must be avoided especially in acute trauma patients. When used, performing endoscopy using CO2 instead of air, is less dangerous in cases of existing or suspected perforations [26, 35, 65].

Nevertheless, ultrasound has not been routinely used in evaluating the diaphragm during the primary and secondary survey in unstable multiple trauma patients. A recent systematic review in children showed that it has a very low sensitivity of 25% (5/20) [66]. It is a tedious study and takes time and possibly it may be useful in tertiary survey when a more detailed study can be done. The distended stomach may hinder its evaluation during the acute setting.

During pregnancy, ultrasound has been reported to be useful for evaluating a suspected DH [67–71]. In pregnant stable patients, MRI should be always performed instead of a CT scan to avoid radiation exposure when a DH is suspected. MRI in pregnant women may be performed on a 1.5 T or 3 T MRI scanner without the administration of an intravenous gadolinium-based contrast agent. In complicated DH, a high signal on T1W, T2W, and T1W fat-saturated sequences indicates ischemic infarction, especially in the strangulated herniated viscera with obstruction or ischemia. DWI may be useful for describing early ischemia [3, 53, 72, 73].

## What is the best treatment for patients with traumatic diaphragmatic hernia?

- Surgery is recommended in stable patients with traumatic DH, preferably with a laparoscopic approach (Strong recommendation based on moderate-quality evidence, 1B)
- In unstable TDH patients, a laparotomy approach is suggested (Weak recommendation based on low-quality evidence, 2C)

## Technical issues

- Sac excision is not suggested (Weak recommendation based on very low-quality evidence, 2D)
- Defect repair using non-absorbable sutures is suggested (Weak recommendation based on low-quality evidence, 2C)
- Mesh use is suggested for defects that cannot be closed with direct suture (Weak recommendation based on low-quality evidence, 2C)
- Biosynthetic, biologic or composite meshes are suggested due to the lower rate of hernia recurrence, higher resistance to infections and lower risk of displacement* (Weak recommendation based on low-quality evidence, 2C)
- Percutaneous endoscopic gastrostomy (PEG), gastrostomy or jejunostomy are suggested in patients with oral intake difficulties (Weak recommendation based on low-quality evidence, 2C)
- Preemptive anti-reflux surgery is not suggested in the emergency traumatic DH setting or complicated hernia (Weak recommendation based on very low-quality evidence, 2D)
- Damage Control Surgery (DCS) is recommended in case of patients with intraoperative instability, hypothermia, coagulopathy, significant acidosis or impossibility to close the diaphragm [74] (Strong recommendation based on low-quality evidence, 1B)

Although there is no consensus or randomized trials on the management and treatment of complicated DH, surgery is the treatment of choice for this pathology [18, 26, 75]. Several studies (meta-analysis, reviews and case series) propose primary repair for the diaphragmatic defects with non-absorbable sutures. This should always be attempted when possible. This is classically done in two layers using interrupted non-absorbable mattress suture. Majority of surgeons still prefer using interrupted non-absorbable 2–0 or 1–0 monofilament or braided sutures in two layers [76–85].

Suturing defects has the benefit of providing a flat surface for placement of the mesh, preventing mesh extrusion through the defect. In a DH with larger defects (> 3 cm), an attempt to primary repair the defect could lead to excessive tension due to the considerable loss of tissue. A very high recurrence rate of 42% has been reported after primary DH repair. In order to reinforce the suture repair, a mesh should be used [83, 86–89].

Excision of the hernial sac is controversial. In most studies, retention of the sac had no obvious complications. In other studies, sac excision occasionally led to pneumomediastinum, damage to pericardium or mediastinal structures. In other studies, sac excision reduced tissue trauma, fluid collection and recurrence, especially in cases where the colon or stomach was contained within the sac because in these cases only the sac was manipulated (rather than its contents) which reduces the possibility of transmural visceral injury or neurovascular injury. This reduced the chance for symptomatic fluid collection since the serous lining membrane was removed. Sac excision also negates the possibility for the remaining sac to act as a lead point for recurrent herniation [90, 91].

Larger defects that cannot be closed primarily should be bridged with a mesh and/or reattach the diaphragm to the ribs by encircling the ribs for fixation, even at a higher rib level than the anatomical one if needed. It was recommended not to perform primary closure of the diaphragm if the distance between the edge of the remaining part of the diaphragm and the chest wall is more than 3–4 cm. Primary diaphragmatic suture or mesh reconstruction can seal the chest cavity without paradoxical chest movement or increase in mortality. Many different materials can be used for the reconstruction, mostly favored by the surgeon’s experience, attitude and preference. The reconstruction is typically performed with synthetic meshes. They are well tolerated, can be bio-prosthetic materials or entirely artificial mesh, either absorbable or non-absorbable [92, 93].

Many reviews have evaluated the role of different meshes in DH repair. A mesh should be used for larger defects. Biological meshes have lower rate of hernia recurrence, higher resistance to infections and lower risk of displacement compared with synthetic meshes [26, 94, 95]. An alternative to biological meshes for the repair, particularly of the clean-contaminated and contaminated diaphragmatic hernia repair, is an absorbable synthetic mesh. Absorbable synthetic mesh has the prospective advantages of reduced cost and minimal constraints in manufacturing alternative sizes. Because of its strength and impermeability, polytetrafluoroethylene (PTFE Gore-Tex™, Gore&Assoc, Arizona, USA) is one of the most common meshes recommended for diaphragmatic reconstruction. It has an advantage of not adhering to the bowel with reduced risk of bowel fistulation [86, 93]. Despite several advantages of using absorbable synthetic mesh, data on diaphragmatic hernia repair in the emergency setting are not available and further studies are warranted [97, 98].

The decision about the best surgical approach in the treatment of diaphragmatic hernias, thoracic or abdominal, depends primarily on the chronicity of the condition, the surgeon’s preferences and skills, and the local resources. Complicated diaphragmatic hernias in unstable patients or those who have signs of strangulation or perforation should be primarily approached through the abdomen, reserving the thoracic approach as a complementary access. Thoracotomy or thoracoscopy may be used, especially in chronic herniation because of the presence of viscero-pleural adhesions and the higher risk of intrathoracic visceral perforation [99–102]. Rarely, a thoraco-abdominal approach for complicated DH has been utilized. This approach can be useful when it is difficult to identify all abdominal injuries or when it is needed to exclude bilateral cavity complications [103–105].

Laparoscopic or thoracoscopic approaches have become the most used approaches to manage complicated DH. Minimally invasive surgery, if properly indicated, is now more feasible, safer, has shorter length of hospital stay, and less morbidity compared with open surgery [89, 106–110]. Robotic surgery of complicated DH repair has been reported only in few cases [111, 112].

Data about robotic DH repair are poor. According to the 2021 WSES position paper on robotic surgery in emergency setting, the potential use of robotic DH repair should be considered by experienced surgical and nursing team in clinically stable patients [113].

Gastrostomy and PEG have added advantage for patients who have difficulties in oral intake. Gastrostomy and PEG are both used to provide fixation of the anterior stomach to the abdominal wall [114–116]. The use of PEG or combined PEG/laparoscopy in the treatment of DH is successful in relieving symptoms, preventing recurrence, stopping progression of complications (such as gastric ischemia), has very low morbidity, and is well tolerated, especially in high-risk elderly patients who are otherwise unsuitable candidates for a definitive surgical repair [117, 118].

Literature data about anti-reflux procedures combined with TDH repair are poorly reported. Anti-reflux procedure, due to the fixation of the organ in the abdominal cavity, may be performed successfully in traumatic diaphragmatic hernia patients with history of gastroesophageal reflux and the need to repair a large defect [26, 109].

In critical patients, Damage Control Surgery (DCS) can be life-saving. In unstable patients with severely injured abdominal organs, a second look may be needed to re-evaluate an ischemic bowel. Furthermore, leaving the abdomen open may prevent abdominal compartment syndrome. Furthermore, DCS should be considered when the diaphragm cannot be closed [119, 120].

## What is the best treatment for patients with non-traumatic diaphragmatic hernia?

- Surgery is recommended in complicated non-traumatic DH (Strong recommendation based on moderate-quality evidence, 2C)
- In unstable non-traumatic complicated DH patients, a laparotomy approach is suggested (Weak recommendation based on low-quality evidence, 2C)
- In stable patients with complicated non-traumatic DH, minimally invasive approach is suggested (Weak recommendation based on very low-quality evidence, 2D)
- Sac excision is not suggested (Weak recommendation based on very low-quality evidence, 2D)
- Defect repair using non-absorbable sutures is suggested (Weak recommendation based on low-quality evidence, 2C)
- Mesh use is suggested especially for larger defects that cannot be closed (Weak recommendation based on low-quality evidence, 2C)
- Biosynthetic, biologic or composite meshes are suggested due to the lower hernia recurrence, higher resistance to infections and lower risk of displacement (Weak recommendation based on low-quality evidence, 2C)
- Gastropexy is suggested in patients with gastric volvulus in order to prevent recurrence (Weak recommendation based on moderate-quality evidence, 2B)
- Percutaneous endoscopic gastrostomy (PEG), gastrostomy or jejunostomy are suggested in patients with preexisting oral intake difficulties (Weak recommendation based on low-quality evidence, 2C)
- Gastrostomy and PEG are suggested to provide fixation of the anterior stomach to the abdominal wall in patients unsuitable or high-risk candidates for a definitive surgical procedure, only in case of non-ischemic stomach (Weak recommendation based on low-quality evidence, 2C)
- Anti-reflux surgery is recommended especially in congenital diaphragmatic hernia repair and in patients with gastroesophageal reflux (Weak recommendation based on low-quality evidence, 2C)
- Toupet or Nissen fundoplication is suggested in case of gastroesophageal disease**.** (Weak recommendation based on low-quality evidence, 2C)
- Thoracoscopy combined with laparoscopy is suggested especially in complicated DH with history of chronic herniation in order to reduce visceral-pleural adhesions and to avoid intrathoracic visceral perforation (Weak recommendation based on very low-quality evidence, 2D)

The treatment of non-traumatic diaphragmatic hernia is similar to traumatic diaphragmatic hernia.

Surgery is the treatment of choice for this condition. [78, 82].

Primary repair for the diaphragmatic defects with non-absorbable sutures should always be attempted when possible [76–85, 121].

However, the treatment of TDH surgeons is reluctant in the use of mesh in the emergency setting due to the risk of infectious complications. In clean-contaminated and contaminated diaphragmatic hernia repair, biologic or most recent biosynthetic meshes can be safely used [94, 96–98].

Several papers advice that the mesh overlaps the defect edge by 1.5–2.5 cm. When tension-free primary closures are difficult, for defects larger than 8 cm or an area of more than 20 cm^2^, the interposition of a graft can be used successfully. Mesh can be fixed using tackers or transfascial sutures. Tackers should be avoided in proximity of the pericardium because of the risk or cardiac complications [109, 122–126]

Minimally invasive abdominal approach can be performed with an excellent safety profile and a reported overall in-hospital mortality rate of 0.14%. Several studies showed similar outcomes in terms of perioperative morbidity and mortality in both laparotomy and minimally invasive approach. In right diaphragmatic hernia, due to the presence of the liver, the diaphragmatic repair can be difficult and a combined or a thoracic approach may be necessary [100, 127–130].

The incidence of gastroesophageal reflux after repair of congenital diaphragmatic hernia is high reaching up to 62%. Accordingly, fundoplication should be performed during congenital hernia [131, 132]. Several studies have reported the role of fundoplication during repair of a sliding hiatal hernia but not for diaphragmatic hernias to manage the associated gastroesophageal reflux. Factors affecting the surgical procedure selection include the history of gastroesophageal reflux, the need to repair a large defect repair, a paraesophageal hernia, or a congenital hernia [133, 134]. In case of gastric volvulus with diaphragmatic hernia gastropexy after detorsion of the volvulus, reduction of herniated structures and repair of the diaphragmatic defect has been successful [136–139].

A Collis procedure or gastric resection is required in selected cases, determined by the gastric condition and should be tailored case by case [100]. Nissen fundoplication is the most common technique performed. Nissen fundoplication and Toupet fundoplication have similar effects on restoration of the mechanical gastroesophageal barrier against gastric reflux. Few papers reported that recurrence rate in Toupet fundoplication is lower compared with Nissen fundoplication [140–145].

## Clinical outcomes

Clinical outcomes following the management of complicated DH are poorly reported in the literature. A wide range of complications are reported with rates varying from 11 to 62.9% [12].

Postoperative pulmonary complications including atelectasis are common following surgery for TDH. Other complications include surgical infection, bleeding, respiratory failure, ileus, gastroesophageal reflux, chronic pain, hernia recurrence, and cardiac injury.

The high morbidity is related to the surgical approach used. In complicated BH, laparoscopic repair has a lower morbidity (6%) compared with the open approach (18%) [14]. The laparoscopic approach of MH repair has similar morbidity rate compared with BH. Laparoscopy approach of MH has a lower morbidity rate (5%) compared with the open approach (17%) [107]. The survival rate in adults presenting with delayed complicated CDH varies between 97 and 100% [146, 147]. The mortality rate in complicated TDH ranges from 14.3 to 20%. [47, 148–150].

The current data regarding recurrence are poor. Hanna et al. reported recurrence in only two cases out of 76 cases after primary repair but only 13 (17.1%) could be followed up [95]. The recurrence of a diaphragmatic hernia may be due to the use of absorbable sutures for the primary repair, suture tension, improper fixation of the prosthetic material to the defect edges with a minimal overlap, increased intra-abdominal pressure due to a prolonged ileus, poor chest toileting and an intra-abdominal sepsis causing tension, disruption of tissue and sutures, leading to mesh displacement or re-opening of the defect. Recurrence is largely due to failure of the host-prosthesis interface, while the synthetic patch integrity is usually maintained [109, 125, 128, 132, 135].

## Conclusions

Complicated diaphragmatic hernia is a rare life-threatening condition. CT scan of the chest and abdomen is the gold standard for diagnosing the diaphragmatic hernia. Laparoscopic repair seems to be the best treatment option for stable patients with complicated diaphragmatic hernias. Open repair is considered necessary in majority of unstable patients in whom Damage Control Surgery can be life-saving.

## Data Availability

The original dataset generated during the current study is available from the corresponding author on reasonable request.

## Abbreviations

DH: Diaphragmatic hernia
CDH: Congenital diaphragmatic hernia
ADH: Acquired diaphragmatic hernia
BH: Bochdalek hernias
MH: Morgagni’s hernia
TDH: Traumatic diaphragmatic hernia
DR: Diaphragmatic rupture

## References

[1] Garne E, Haeusler M, Barisic I, Gjergja R, Stoll C, Clementi M (2002). Congenital diaphragmatic hernia: evaluation of prenatal diagnosis in 20 European regions. Ultrasound Obstet Gynecol.

[2] Baerg J, Kanthimathinathan V, Gollin G (2012). Late-presenting congenital diaphragmatic hernia: diagnostic pitfalls and outcome. Hernia.

[3] Eren S, Ciris F (2005). Diaphragmatic hernia: diagnostic approaches with review of the literature. Eur J Radiol.

[4] Meyers BF, McCabe CJ (1993). Traumatic diaphragmatic hernia. Occult marker of serious injury. Ann Surg.

[5] Díaz Candelas DA, de la Plaza LR, Arteaga Peralta V, Ramia JM (2020). Hernia diafragmática complicada. Cir Esp.

[6] D'Orio V, Demondion P, Lebreton G, Coutance G, Varnous S, Leprince P (2017). Acquired transdiaphragmatic hernia: an unusual cause of cardiac tamponade. Asian Cardiovasc Thorac Ann.

[7] Lerner CA, Dang H, Kutilek RA (1997). Strangulated traumatic diaphragmatic hernia simulating a subphrenic abscess. J Emerg Med.

[8] Guyatt GH, Oxman AD, Vist GE, Kunz R, Falck-Ytter Y, Alonso-Coello P (2008). GRADE: an emerging consensus on rating quality of evidence and strength of recommendations. BMJ.

[9] Brozek JL, Akl EA, Jaeschke R, Lang DM, Bossuyt P, Glasziou P (2009). Grading quality of evidence and strength of recommendations in clinical practice guidelines: part 2 of 3. The GRADE approach to grading quality of evidence about diagnostic tests and strategies. Allergy.

[10] Mullins ME, Stein J, Saini SS (2001). Prevalence of incidental Bochdalek’s hernia in a large adult population. AJR Am J Roentgenol.

[11] Deprest J, Brady P, Nicolaides K, Benachi A, Berg C, Vermeesch J, Gardener G, Gratacos E (2014). Prenatal management of the fetus with isolated congenital diaphragmatic hernia in the era of the TOTAL trial. Semin Fetal Neonatal Med.

[12] Hosgor M, Karaca I, Karkiner A (2004). Associated malformations in delayed presentation of congenital diaphragmatic hernia. J Pediatr Surg.

[13] Salacin S, Alper B, Cekin N, Gulmen MK (1994). Bochdalek hernia in adulthood: a review and an autopsy case report. J Forensic Sci.

[14] Brown SR, Horton JD, Trivette E, Hofmann LJ, Johnson JM (2011). Bochdalek hernia in the adult: demographics, presentation, and surgical management. Hernia.

[15] Swain JM, Klaus A, Achem SR, Hinder RA (2001). Congenital diaphragmatic hernia in adults. Surg Innov.

[16] Mullins ME, Saini S (2005). Imaging of incidental Bochdalek hernia. Semin Ultrasound CT MRI.

[17] Maish MS (2010). The diaphragm. Surg Clin N Am.

[18] Nguyen T, Eubanks PJ, Nguyen D, Klein SR (1998). The laparoscopic approach for repair of Morgagni hernias. Jsls.

[19] Kitano Y, Lally KP, Lally PA (2005). Late-presenting congenital diaphragmatic hernia. J Pediatr Surg.

[20] Minneci PC, Deans KJ, Kim P, Mathisen DJ (2004). Foramen of Morgagni hernia: changes in diagnosis and treatment. Ann Thorac Surg.

[21] Deslauriers J (1998). Eventration of the diaphragm. Chest Surg Clin N Am.

[22] Haino K, Serikawa T, Itsukaichi M (2011). Morgagni hernia with massive pericardial effusion diagnosed in the second trimester: prenatal diagnosis and perinatal management. Fetal Diagn Ther.

[23] Kahrilas PJ, Kim HC, Pandolfino JE (2008). Approaches to the diagnosis and grading of hiatal hernia. Best Pract Res Clin Gastroenterol.

[24] Skinner DB, Berk JE (1985). Chapter 53. Hernias. Gastroenterology.

[25] Rubikas R (2001). Diaphragmatic injuries. Eur J Cardiothorac Surg.

[26] Testini M, Girardi A, Isernia RM (2017). Emergency surgery due to diaphragmatic hernia: case series and review. World J Emerg Surg.

[27] Lu J, Wang B, Che X (2016). Delayed traumatic diaphragmatic hernia: a case-series report and literature review. Medicine.

[28] Panda A, Kumar A, Gamanagatti S, Patil A, Kumar S, Gupta A (2014). Traumatic diaphragmatic injury: a review of CT signs and the difference between blunt and penetrating injury. Diagn Interv Radiol.

[29] Lomoschitz FM, Eisenhuber E, Linnau KF, Peloschek P, Schoder M, Bankier AA (2003). Imaging of chest trauma: radiological patterns of injury and diagnostic algorithms. Eur J Radiol.

[30] Bergeron E, Clas D, Ratte S (2002). Impact of deferred treatment of blunt diaphragmatic rupture: a 15-year experience in six trauma centers in Quebec. J Trauma.

[31] Zantut LF, Machado MA, Volpe P, Poggetti RS, Birolini D (1993). Bilateral diaphragmatic injury diagnosed by laparoscopy. Rev Paul Med.

[32] Brown GL, Richardson JD (1985). Traumatic diaphragmatic hernia: a continuing challenge. Ann Thorac Surg.

[33] Aun F, Lourenção JL, Younes RN, Alfieri Júnior F, Birolini D, Oliveira MR (1982). Contribuição ao estudo da história natural e dos fatores de risco das hérnias diafragmáticas traumáticas. Rev Hosp Clin Fac Med Univ São Paulo.

[34] Moore EE, Malangoni MA, Cogbill TH, Shackford SR, Champion HR, Jurkovich GJ, McAninch JW, Trafton PG (1994). Organ injury scaling. IV: thoracic vascular, lung, cardiac, and diaphragm. J Trauma.

[35] Nayak HK, Maurya G, Kapoor N, Kar P. Delayed presentation of congenital diaphragmatic hernia presenting with intrathoracic gastric volvulus: a case report and review. BMJ Case Rep. 2012;2012. 10.1136/bcr-2012-007332 PMC4544210 23195824

[36] Cullen ML, Klein MD, Philippart AI (1985). Congenital diaphragmatic hernia. Surg Clin N Am.

[37] Zani A, Chung WK, Deprest J (2022). Congenital diaphragmatic hernia. Nat Rev Dis Primers.

[38] Bujanda L, Larrucea I, Ramos F, Munoz C, Sanchez A, Fernandez I (2001). Bochdalek’s hernia in adults. J Clin Gastroenterol.

[39] Rowell SE, Barbosa RR, Diggs BS (2011). Specific abbreviated injury scale values are responsible for the underestimation of mortality in penetrating trauma patients by the injury severity score. J Trauma.

[40] Shah R, Sabanathan S, Mearns AJ, Choudhury AK (1995). Traumatic rupture of diaphragm. Ann Thorac Surg.

[41] Raitio A, Salim A, Losty PD (2021). Congenital diaphragmatic hernia-does the presence of a hernia sac improve outcome? A systematic review of published studies. Eur J Pediatr.

[42] Mihos P, Potaris K, Gakidis J (2003). Traumatic rupture of the diaphragm: experience with 65 patients. Injury.

[43] Murray JA, Demetriades D, Asensio JA, Cornwell EE, Velmahos GC, Belzberg H (1998). Occult injuries to the diaphragm: prospective evaluation of laparoscopy in penetrating injuries to the left lower chest. J Am Coll Surg.

[44] Grimes OF (1974). Traumatic injuries of the diaphragm. Diaphragmatic hernia Am J Surg.

[45] Blackstone MM, Mistry RD (2007). Late-presenting congenital diaphragmatic hernia mimicking bronchiolitis. Pediatr Emerg Care.

[46] Basol O, Bilge H (2022). Our surgical experience in traumatic and congenital diaphragmatic hernia: Single-center study. Niger J Clin Pract.

[47] Peer SM, Devaraddeppa PM, Buggi S (2009). Traumatic diaphragmatic hernia-our experience. Int J Surg.

[48] Wu CT, Huang JL, Hsia SH, Lin JJ, Lai SH (2009). Late-presenting congenital diaphragmatic hernia in pediatric emergency room: two case reports. Eur J Pediatr.

[49] Miller L, Bennett EV, Root HD, Trinkle JK, Grover FL (1984). Management of penetrating and blunt diaphragmatic injury. J Trauma.

[50] Demetriades D, Kakoyiannis S, Parekh D, Hatzitheofilou C (1988). Penetrating injuries of the diaphragm. Brit J Surg.

[51] Sangster G, Ventura VP, Carbo A, Gates T, Garayburu J, D’Agostino H (2007). Diaphragmatic rupture: a frequently missed injury in blunt thoracoabdominal trauma patients. Emerg Radiol.

[52] Hirano ES, Silva VG, Bortoto JB, Barros RHO, Caserta NMG, Fraga GP. Plain chest radiographs for the diagnosis of post-traumatic diaphragmatic hernia. Rev Col Bras Cir. 2012; 39(4). Available at http://www.scielo.br/rcbc 10.1590/s0100-69912012000400007 22936226

[53] Kurniawan N, Verheyen L, Ceulemans J (2013). Acute chest pain while exercising: a case report of Bochdalek hernia in an adolescent. Acta Chir Belg.

[54] Magu S, Agarwal S, Singla S (2012). Computed tomography in the evaluation of diaphragmatic hernia following blunt trauma. Indian J Surg.

[55] Desser TS, Edwards B, Hunt S, Rosenberg J, Purtill MA, Jeffrey RB (2010). The dangling diaphragm sign: sensitivity and comparison with existing CT signs of blunt traumatic diaphragmatic rupture. Emerg Radiol.

[56] Cantwell CP (2006). The dependent viscera sign. Radiology.

[57] Rees O, Mirvis SE, Shanmuganathan K (2005). Multidetector-row CT of right hemidiaphragmatic rupture caused by blunt trauma: a review of 12 cases. Clin Radiol.

[58] Hammer MM, Raptis DA, Mellnick VM, Bhalla S, Raptis CA (2017). Traumatic injuries of the diaphragm: overview of imaging findings and diagnosis. Abdom Radiol (NY).

[59] Polireddy K, Hoff C, Kinger NP, Tran A, Maddu K (2022). Blunt thoracic trauma: role of chest radiography and comparison with CT - findings and literature review. Emerg Radiol.

[60] Paes FM, Durso AM, Danton G, Castellon I, Munera F (2020). Imaging evaluation of diaphragmatic injuries: Improving interpretation accuracy. Eur J Radiol.

[61] Kaur R, Prabhakar A, Kochhar S, Dalal U (2015). Blunt traumatic diaphragmatic hernia: pictorial review of CT signs. Indian J Radiol Imaging.

[62] Zantut LF, Ivatury RR, Smith RS (1997). Diagnostic and therapeutic laparoscopy for penetrating abdominal trauma: a multicenter experience. J Trauma.

[63] Ivatury RR, Simon RJ, Stahl WM (1993). A critical evaluation of laparoscopy in penetrating abdominal trauma. J Trauma.

[64] Ivatury RR (2005). Laparoscopy and thoracoscopy in penetrating thoracoabdominal injuries. Eur Surg.

[65] Endoscopic management of gastrointestinal motility disorders—part 2: European Society of Gastrointestinal Endoscopy (ESGE) Guideline. Bas L. A. M. Weusten et al. 2020. https://www.esge.com/assets/downloads/pdfs/guidelines/2020_a_1171_3174.pdf 10.1055/a-1171-3174 32462649

[66] Theodorou CM, Jackson JE, Beres AL, Leshikar DE (2021). Blunt traumatic diaphragmatic hernia in children: a systematic review. J Surg Res.

[67] Genc MR, Clancy TE, Ferzoco SJ, Norwitz E (2003). Maternal congenital diaphragmatic hernia complicating pregnancy. Obstet Gynecol.

[68] Julien F, Drolet S, Lévesque I, Bouchard A (2011). The right lateral position for laparoscopic diaphragmatic hernia repair in pregnancy: technique and review of the literature. J Laparoendosc Adv Surg Tech A.

[69] Ngai I, Sheen J-J, Govindappagari S, Garry DJ. Bochdalek hernia in pregnancy. Case Rep. 2012; 2012, bcr2012006859. 10.1136/bcr-2012-006859 PMC4544797 22967686

[70] Vasquez DN, Basualdo MN, Aphalo VM, Carreras LP, Plotnikow GA, Intile AD, Moreira J (2019). Complications of congenital hernia in pregnancy: a case report. A A Pract.

[71] Suhardja TS, Vaska A, Foley D, Gribbin J (2019). Adult Bochdalek hernia in a pregnant woman. ANZ J Surg.

[72] Genc MR, Clancy TE, Ferzoco SJ, Norwitz E (2003). Maternal congenital diaphragmatic hernia complicating pregnancy. Obstet Gynecol.

[73] Ngai I, Sheen JJ, Govindappagari S, Garry DJ. Bochdalek hernia in pregnancy. BMJ Case Rep. 2012;2012. 10.1136/bcr-2012-006859 PMC4544797 22967686

[74] Coccolini F, Roberts D, Ansaloni L (2018). The open abdomen in trauma and non-trauma patients: WSES guidelines. World J Emerg Surg.

[75] Grillo IA, Jastaniah SA, Bayoumi AH (2000). Traumatic diaphragmatic hernia: an Asir region (Saudi Arabia) experience. Indian J Chest Dis Allied Sci.

[76] Okyere I, Okyere P, Glover PSK (2019). Traumatic right diaphragmatic rupture with hepatothorax in Ghana: two rare cases. Pan Afr Med J.

[77] Mittal A, Pardasani M, Baral S, Thakur S (2018). A rare case report of Morgagni Hernia with Organo-axial gastric volvulus and concomitant Para-esophageal hernia, repaired laparoscopically in a Septuagenarian. Int J Surg Case Rep.

[78] Sathasivam R, Bussa G, Viswanath Y, Obuobi RB, Gill T, Reddy A, Shanmugam V, Gilliam A, Thambi P (2019). ‘Mesh hiatal hernioplasty’ versus ‘suture cruroplasty’ in laparoscopic para-oesophageal hernia surgery; a systematic review and meta-analysis. Asian J Surg.

[79] Bona D, Lombardo F, Matsushima K, Cavalli M, Panizzo V, Mendogni P, Bonitta G, Campanelli G, Aiolfi A (2021). Diaphragmatic herniation after esophagogastric surgery: systematic review and meta-analysis. Langenbecks Arch Surg.

[80] Koch O, von Rahden B, Wykypiel H, Schoppmann SF, Függer R, Rosanelli G, Emmanuel K, Weitzendorfer M (2021). Planning and design of a prospective randomised multi-centre trial on the repair of large hiatal Hernias with sutures vs. Pledgeted sutures vs. absorbable mesh. Zentralbl Chir.

[81] Analatos A, Håkanson BS, Lundell L, Lindblad M, Thorell A (2020). Tension-free mesh versus suture-alone cruroplasty in antireflux surgery: a randomized, double-blind clinical trial. Br J Surg.

[82] Watson DI, Thompson SK, Devitt PG, Aly A, Irvine T, Woods SD, Gan S, Game PA, Jamieson GG (2020). Five year follow-up of a randomized controlled trial of laparoscopic repair of very large Hiatus hernia with sutures versus absorbable versus nonabsorbable mesh. Ann Surg.

[83] Filosso PL, Guerrera F, Sandri A, Lausi PO, Lyberis P, Bora G, Roffinella M, Ruffini E (2019). Surgical management of chronic diaphragmatic hernias. J Thorac Dis.

[84] Gerdes S, Vetter D, Müller PC, Kapp JR, Gutschow CA (2021). Current surgical concepts for type III hiatal hernia: a survey among members of the Swiss Society of Visceral Surgery. Swiss Med Wkly.

[85] Oor JE, Roks DJ, Koetje JH (2018). Randomized clinical trial comparing laparoscopic hiatal hernia repair using sutures versus sutures reinforced with non-absorbable mesh. Surg Endosc.

[86] Shao G, Wu L, Li J, Dai C (2020). Laparoscopic diaphragmatic hernia repair with mesh reinforcement. Am Surg.

[87] Palanivelu C, Rangarajan M, Rajapandian S, Amar V, Parthasarathi R (2009). Laparoscopic repair of adult diaphragmatic hernias and eventration with primary sutured closure and prosthetic reinforcement: a retrospective study. Surg Endosc.

[88] Choy KT, Chiam HC. Dyspnoea and constipation: rare case of large bowel obstruction secondary to an incarcerated Morgagni hernia. BMJ Case Rep. 2019;12(6). 10.1136/bcr-2019-229507 PMC6557358 31177195

[89] Mohamed M, Al-Hillan A, Shah J, Zurkovsky E, Asif A, Hossain M (2020). Symptomatic congenital Morgagni hernia presenting as a chest pain: a case report. J Med Case Rep.

[90] Loong TP, Kocher HM (2005). Clinical presentation and operative repair of hernia of Morgagni. Postgrad Med J.

[91] Godazandeh G, Mokhtari-esbuie F (2016). Laparoscopic repair of Morgagni hernia: three-case presentation and the literature. Case Rep Surg.

[92] Solli P, Bertolaccini L, Brandolini J, Pardolesi A. Reconstructive techniques after diaphragm resection and use of the diaphragmatic flap in thoracic surgery. Shanghai Chest. 2017; 1, 4

[93] Kuwahara H, Salo J, Tukiainen E (2018). Diaphragm reconstruction combined with thoraco-abdominal wall reconstruction after tumor resection. J Plast Surg Hand Surg.

[94] Antoniou SA, Pointner R, Granderath FA, Köckerling F (2015). the use of biological meshes in diaphragmatic defects —an evidence-based review of the literature. Front Surg.

[95] Coccolini F, Agresta F, Bassi A (2012). Italian biological prosthesis work-group (IBPWG): proposal for a decisional model in using biological prosthesis. World J Emerg Surg.

[96] Jee Y (2017). Laparoscopic diaphragmatic hernia repair using expanded polytetrafluoroethylene (ePTFE) for delayed traumatic diaphragmatic hernia. Wideochir Inne Tech Maloinwazyjne.

[97] Panici Tonucci T, Asti E, Sironi A, Ferrari D, Bonavina L (2020). Safety and efficacy of crura augmentation with Phasix ST mesh for large Hiatal hernia: 3-year single-center experience. J Laparoendosc Adv Surg Tech A.

[98] Asti E, Sironi A, Bonitta G, Lovece A, Milito P, Bonavina L (2017). Crura augmentation with Bio-A® mesh for laparoscopic repair of hiatal hernia: single-institution experience with 100 consecutive patients. Hernia J Hernias Abdom Wall Surg.

[99] Mansour KA (1997). Trauma to the diaphragm. Chest Surg Clin N Am.

[100] Katsaros I, Katelani S, Giannopoulos S, Machairas N, Kykalos S, Koliakos N, Kapetanakis EI, Bakopoulos A, Schizas D (2021). Management of Morgagni’s hernia in the adult population: a systematic review of the literature. World J Surg.

[101] Zheng Z, Liu X, Xin C, Zhang W, Gao Y, Zeng N, Li M, Cai J, Meng F, Liu D, Zhang J, Yin J, Zhang J, Zhang Z (2021). A new technique for treating hiatal hernia with gastroesophageal reflux disease: the laparoscopic total left-side surgical approach. BMC Surg.

[102] Walker R, Wiggins T, Blencowe NS, Findlay JM, Wilson M, Currie AC, Hornby S, Markar SR, Rahman S, Lloyd M, Hollyman M, Jaunoo S, ARROW Study Group (2021). A multicenter prospective audit to investigate the current management of patients undergoing anti-reflux surgery in the UK: audit & review of anti-reflux operations & workup. Dis Esophagus Off J Int Soc Dis Esophagus.

[103] Nursal TZ, Ugurlu M, Kologlu M, Hamaloglu E (2001). Traumatic diaphragmatic hernias: a report of 26 cases. Hernia.

[104] Massloom HS (2016). Acute bowel obstruction in a giant recurrent right Bochdalek’s hernia: a report of complication on both sides of the diaphragm. N Am J Med Sci.

[105] Barbetakis N, Efstathiou A, Vassiliadis M, Xenikakis T, Fessatidis I (2006). Bochdaleck's hernia complicating pregnancy: case report. World J Gastroenterol.

[106] Rehman A, Maliyakkal AM, Naushad VA, Allam H, Suliman AM (2018). A lady with severe abdominal pain following a Zumba dance session: a rare presentation of Bochdalek hernia. Cureus.

[107] Horton JD, Hofmann LJ, Hetz SP (2008). Presentation and management of Morgagni hernias in adults: a review of 298 cases. Surg Endosc.

[108] Susmallian S, Raziel A (2017). A rare case of Bochdalek hernia with concomitant para-Esophageal hernia, repaired laparoscopically in an octogenarian. Am J Case Rep.

[109] Perrone G, Giuffrida M, Annicchiarico A (2020). Complicated diaphragmatic hernia in emergency surgery: systematic review of the literature. World J Surg.

[110] Ceccarelli G, Pasculli A, Bugiantella W (2020). Minimally invasive laparoscopic and robot-assisted emergency treatment of strangulated giant hiatal hernias: report of five cases and literature review. World J Emerg Surg.

[111] Jambhekar A, Robinson S, Housman B, Nguyen J, Gu K, Nakhamiyayev V (2018). Robotic repair of a right-sided Bochdalek hernia: a case report and literature review. J Robot Surg.

[112] Hunter LM, Mozer AB, Anciano CJ, Oliver AL, Iannettoni MD, Speicher JE (2019). Robotic-assisted thoracoscopic repair of right-sided Bochdalek hernia in adults: a two-case series. Innovations (Phila).

[113] Deangelis, N., Khan, J., Marchegiani, F. et al. Robotic surgery in emergency setting: 2021 WSES position paper. World J Emerg Surg. 2022;17,4. 10.1186/s13017-022-00410-6] 10.1186/s13017-022-00410-6 PMC8781145 35057836

[114] Baudet JS, Ammengol-Miro JR, Medin C, Accarino AM, Vilaceca J, Malagelada JR (1997). Percutaneous endoscopic gastrostomy as a treatment for chronic gastric volvulus. Endoscopy.

[115] Golash V (2006). Laparoscopic reduction of acute intrathoracic herniation of colon, omentum and gastric volvulus. J Minim Access Surg.

[116] Ladiwala ZFR, Sheikh R, Ahmed A, Zahid I, Memon AS (2018). Gastric volvulus through Morgagni hernia and intestinal diverticulosis in an adult patient: a case report. BMC Surg.

[117] Kercher KW, Matthews BD, Ponsky JL (2001). Minimally invasive management of paraesophageal herniation in the high-risk surgical patient. Am J Surg.

[118] Xenos ES (2000). Percutaneous endoscopic gastrostomy in a patient with a large hiatal hernia using laparoscopy. JSLS.

[119] Coccolini F, Biffl W, Catena F (2015). The open abdomen, indications, management and definitive closure. World J Emerg Surg.

[120] He S, Sade I, Lombardo G, Prabhakaran K. Acute presentation of congenital diaphragmatic hernia requiring damage control laparotomy in an adult patient. J Surg Case Rep. 2017; 2017: rjx144. 10.1093/jscr/rjx144 PMC5534014 28775839

[121] Arevalo G, Harris K, Sadiq A, Calin ML, Nasri B, Singh K (2017). Repair of Morgagni hernia in adults with primary closure and mesh placement: first robotic experience. J Laparoendosc Adv Surg Tech A.

[122] Yavuz N, Yiğitbasi R, Sunamak O, As A, Oral C, Erguney S (2006). Laparoscopic repair of Morgagni. Surg Laparosc Endosc Percutan Tech.

[123] Thoman DS, Hui T, Phillips EH (2002). Laparoscopic diaphragmatic hernia repair. Surg Endosc.

[124] Percivale A, Stella M, Durante V (2005). Laparoscopic treatment of Morgagni-Larrey hernia: technical details and report of a series. J Laparoendosc Adv Surg Tech A.

[125] Lim L, Gilyard SM, Sydorak RM, Lau ST, Yoo EY, Shaul DB (2019). Minimally invasive repair of pediatric Morgagni hernias using transfascial sutures with extracorporeal knot tying. Perm J.

[126] Köckerling F, Schug-Pass C, Bittner R (2018). A word of caution: never use tacks for mesh fixation to the diaphragm!. Surg Endosc.

[127] Whealon MD, Blondet JJ, Gahagan JV, Phelan MJ, Nguyen NT (2017). Volume and outcomes relationship in laparoscopic diaphragmatic hernia repair. Surg Endosc.

[128] McDonald AA, Robinson BRH, Alarcon L (2018). Evaluation and management of traumatic diaphragmatic injuries: a practice management guideline from the eastern association for the surgery of trauma. J Trauma Acute Care Surg.

[129] Guner A, Ozkan OF, Bekar Y, Kece C, Kaya U, Reis E (2012). Management of delayed presentation of a right-side traumatic diaphragmatic rupture. World J Surg.

[130] Young MC, Saddoughi SA, Aho JM (2019). Comparison of laparoscopic versus open surgical management of Morgagni hernia. Ann Thorac Surg.

[131] Koot VC, Bergmeijer JH, Bos AP, Molenaar JC (1993). Incidence and management of gastroesophageal reflux after repair of congenital diaphragmatic hernia. J Pediatr Surg.

[132] Asti E, Lovece A, Bonavina L, Milito P, Sironi A, Bonitta G, Siboni S (2016). Laparoscopic management of large hiatus hernia: five-year cohort study and comparison of mesh-augmented versus standard crura repair. Surg Endosc.

[133] Kasalický M, Koblihová E (2015). Chirurgie hiátové kýly a refluxní choroby jícnu, Nissen, nebo Toupet? [Surgery of the hiatal hernia and gastroesophageal reflux dinase, Nissen or Toupet?]. Rozhl Chir.

[134] Mittal SK, Bikhchandani J, Gurney O, Yano F, Lee T (2011). Outcomes after repair of the intrathoracic stomach: objective follow-up of up to 5 years. Surg Endosc.

[135] Ayala JA, Naik-Mathuria B, Olutoye OO (2008). Delayed presentation of congenital diaphragmatic hernia manifesting as combined-type acute gastric volvulus: a case report and review of the literature. J Pediatr Surg.

[136] McIntyre RC, Bensard DD, Karrer FM, Hall RJ, Lilly JR (1994). The pediatric diaphragm in acute gastric volvulus. J Am Coll Surg.

[137] Kotobi H, Auber F, Otta E, Meyer N, Audry G, Hélardot PG (2005). Acute mesenteroaxial gastric volvulus and congenital diaphragmatic hernia. Pediatr Surg Int.

[138] Gerstle JT, Chiu P, Emil S (2009). Gastric volvulus in children: lessons learned from delayed diagnoses. Semin Pediatr Surg.

[139] Pérez-Egido L, Parente A, Cerdá JA (2015). Acute gastric volvulus and congenital diaphragmatic hernia, case report and review. Afr J Paediatr Surg.

[140] Shehzad K, Askari A, Slesser AAP, Riaz A (2019). A safe and effective technique of paraesophageal hernia reduction using combined laparoscopy and nonsutured PEG gastropexy in high-risk patients. JSLS.

[141] Kieffer J, Sapin E, Berg A, Beaudoin S, Bargy F, Helardot PG (1995). Gastroesophageal reflux after repair of congenital diaphragmatic hernia. J Pediatr Surg.

[142] Nagaya M, Akatsuka H, Kato J (1994). Gastroesophageal reflux occurring after repair of congenital diaphragmatic hernia. J Pediatr Surg.

[143] Verbelen T, Lerut T, Coosemans W (2013). Antireflux surgery after congenital diaphragmatic hernia repair: a plea for a tailored approach. Eur J Cardiothorac Surg.

[144] Shea B, Boyan W, Decker J, Almagno V, Binenbaum S, Matharoo G, et al. Emergent repair of paraesophageal hernias and the argument for elective repair. JSLS. 2019;23. 10.4293/JSLS.2019.00015 PMC6600053 31285652

[145] Köhler G, Koch OO, Antoniou SA, Emmanuel K, Pointner R (2015). “Acute intrathoracic stomach!” how should we deal with complicated type IV paraesophageal hernias?. Hernia.

[146] Kim JM, Couluris M, Schnapf BM. Late-presenting left-sided morgagni congenital diaphragmatic hernia in a 9-year-old male. ISRN Pulmonol. 2011.

[147] Downard CD, Jaksic T, Garza JJ (2003). Analysis of an improved survival rate for congenital diaphragmatic hernia. J Pediatr Surg.

[148] Thiam O, Konate I, Gueye ML (2016). Traumatic diaphragmatic injuries: epidemiological, diagnostic and therapeutic aspects. Springerplus.

[149] Chughtai T, Ali S, Sharkey P, Lins M, Rizoli S (2009). Update on managing diaphragmatic rupture in blunt trauma: a review of 208 consecutive cases. Can J Surg.

[150] Kruger VF, Calderan TRA, de Carvalho RB, Hirano ES, Fraga GP (2022). Never to be missed again—an analysis of 55 consecutive cases of traumatic diaphragmatic hernia. S Afr J Surg.

